# Application of next generation sequencing to CEPH cell lines to discover variants associated with FDA approved chemotherapeutics

**DOI:** 10.1186/1756-0500-7-360

**Published:** 2014-06-12

**Authors:** Gunjan D Hariani, Ernest J Lam, Tammy Havener, Pui-Yan Kwok, Howard L McLeod, Michael J Wagner, Alison A Motsinger-Reif

**Affiliations:** 1 Bioinformatics Research Center, North Carolina State University, 307 Ricks Hall, 1 Lampe Dr, Raleigh, NC 27695 CB7566, USA; 2 Department of Statistics, North Carolina State University, Raleigh, NC, USA; 3 Cardiovascular Research Institute, UCSF School of Medicine, San Francisco, CA, USA; 4 Center for Institute of Pharmacogenomics and Individualized Therapy, UNC Chapel Hill, Chapel Hill, NC, USA; 5 Moffitt Cancer Center, Tampa, FL, USA

**Keywords:** Cytotoxicity, Next generation sequencing, Lymphoblastoid cell lines, Pharmacogenomics, Candidate gene

## Abstract

**Background:**

The goal of this study was to perform candidate gene association with cytotoxicity of chemotherapeutics in cell line models through resequencing and discovery of rare and low frequency variants along with common variations. Here, an association study of cytotoxicity response to 30 FDA approved drugs was conducted and we applied next generation targeted sequencing technology to discover variants from 103 candidate genes in 95 lymphoblastoid cell lines from 14 CEPH pedigrees. In this article, we called variants across 95 cell lines and performed association analysis for cytotoxic response using the Family Based Association Testing method and software.

**Results:**

We called 2281 variable SNP genotypes across the 103 genes for these cell lines and identified three genes of significant association within this marker set. Specifically, ATP-binding cassette, sub-family C, member 5 (*ABCC5*), metallothionein 1A (*MT1A*) and NAD(P)H dehydrogenase quinone1 (*NQO1*) were significantly associated with oxaliplatin drug response. The significant SNP on *NQO1* (rs1800566) has been linked with poor survival rates in patients with non-small cell lung cancer treated with cisplatin (which belongs to the same class of drugs as oxaliplatin). A SNP (rs1846692) near the 5′ region of *MT1A* was associated with arsenic trioxide.

**Conclusions:**

The results from this study are promising and this serves as a proof-of-principle demonstration of the use of sequencing data in the cytotoxicity models of human cell lines. With increased sample sizes, such studies will be a fast and powerful way to associate common and rare variants with drug response; while overcoming the cost and time limitations to recruit cohorts for association study.

## Background

There is increasing evidence that genetic variation can explain some inter-individual variation in efficacy and toxicity across a spectrum of drugs used for treatment of various diseases. In situations where genetic factors dictate or at least predict drug response, individualized therapy holds a lot of promise and has already shown success in drug choice and dosing [1]. A lot of these influential genetic factors have been identified in genetic association studies nested within clinical trials, but these studies are generally difficult to conduct with high power due to difficulty in cohort recruitment, sample size limitations, phenotype characterization, time and cost factors, etc. [2,3].

An alternate approach that has recently emerged to conducting candidate gene studies in clinical subjects is to use well-established cell lines (e.g. lymphoblastoid cell lines (LCL)). Drug phenotyping is done on these cell lines, and natural genetic variation in these cell lines is tested for association in a discovery phase. Of course the use of cell line based model has its own limitations too – phenotypes may not completely reflect observed human phenotypes, not all enzymes involved in a drug response may be produced by the cell line and the choice of cell line for a study may bias the results. Despite these limitations, there are a large number of studies that have demonstrated that the LCL model can be an effective and efficient way to identify new potential drug-response associated genes. For a review of cell line models in pharmacogenomics, including a full discussion of their advantages, disadvantages, and successes, see [4]. Recent LCL studies have shown that this model can be used to estimate heritability of dose response [5], and it has been used to recapitulate known drug response genes [6].

These previous studies have been performed using single nucleotide polymorphisms (SNPs). To our knowledge, such mapping has never been performed using next generation sequencing. In the current study, we use next generation sequencing in a candidate gene approach to perform an association study of cytotoxic response for 30 chemotherapy drugs. Response to each of these drugs had previously been shown to have a heritable component [5], motivating the association mapping performed in the current study. We used a candidate gene approach, with more than 100 candidate genes that were targeted for sequencing in 95 lymphoblastoid cell lines derived from the Centre d’Etude du Polymorphisme Humain (CEPH) population [7]. These cell lines were derived from individuals from 14 pedigrees.

Because the individuals were from related pedigrees, family based association testing was performed via the FBAT software [8]. After a multiple hypothesis correction was applied to each drug phenotype independently, we found statistically significant hits for two drugs (oxaliplatin and arsenic trioxide) and some of the significant markers identified in this study have been previously implicated with drug response. This study demonstrates the potential of using resequencing in cell-line cytotoxicity models of drug response, and proposed new candidate genes that may be associated with drug response.

## Results and discussion

After variant calling was performed after a careful calling pipeline and series of quality control screens, a total of 2281 SNPs were called across all samples. Of those 2281 SNPs, 904 (39.6%) are low frequency SNPs – they were found in less than 5% of the samples used in this study. A majority of these SNPs are intronic (46.9%), 20.24% are exonic, and 18.8% are present in UTR. The remaining 14.06% of SNPs did not have a transcript definition associated with them for annotation. All the 2281 SNPs were tested for association with the dose response phenotypes derived from cytotoxicity experiments for 30 drugs, where the phenotype used for association analysis was a measure of cell viability after treatment with different doses of each of the 30 drugs. The drugs evaluated are listed in Table 1. The 2281 SNPs were tested for association using the Family Based Association Testing (FBAT) analysis tools, and an FDR correction for each drug was performed such that q = 0.05 was used to ascribe significance [9].

**Table 1:** List of 30 drugs used in this study

**Drug**	**Class**
Busulfan	Alkyl sulfonate
Mitoxantrone	Anthracenediones
Daunorubicin	Anthracyclines
Doxorubicin	Anthracyclines
Epirubicin	Anthracyclines
Idarubicin	Anthracyclines
Topotecan	Camptotheca
Hydroxyurea	deoxyribonucleotide
Trichostatin A	histone deacetylase inhibitor
Rapamycin	mTOR inhibitor
Carboplatin	Platinum
Oxaliplatin	Platinum
Etoposide	Podophyllum
Teniposide	Podophyllum
Cladribine	Purine
Fludarabine	Purine
5-fluorouracil	Pyrimidine
Azacitidine	Pyrimidine
Cytarabine	Pyrimidine
Floxuridine	Pyrimidine
Gemcitabine	Pyrimidine
Bleomycin	Streptomyces
Mitomycin	Streptomyces
Docetaxel	Taxane
Paclitaxel	Taxane
Temozolomide	Triazines
Vinblastine	Vinca alkaloid
Vincristine	Vinca alkaloid
Vinorelbine	Vinca alkaloid
Arsenic trioxide	Other

The drugs and their chemical structure/functional class are listed.

**Figure 1:**
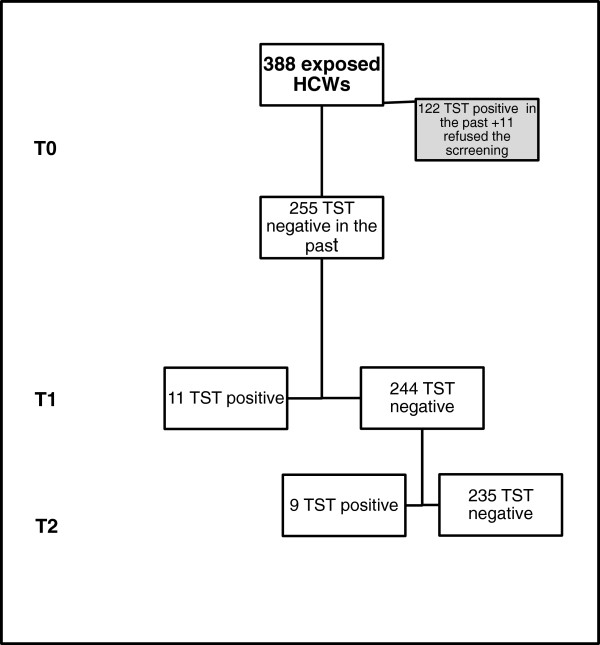
**Manhattan plot for association with oxaliplatin.** Manhattan plot for oxaliplatin is shown. The markers in black are significantly associated at a FDR corrected threshold of 0.05.

Our results show that two drugs - Arsenic Trioxide and Oxaliplatin - had statistically significant hits after controlling for multiple testing. A Manhattan plot for the association results for Oxaliplatin is shown in Figure 1. Manhattan plots for all association results across the candidate genes for all 30 drugs are shown in Additional file 1: Figure S1. Details of the significant SNPs can be found in Table 2.

**Table 2:** Significant hits from association

**Drug(s)**	**SNP ID**	**SNP position**	**Gene**	**q-value**	**MAF**
Arsenic Trioxide	rs60900828 (A/T)	Chr16:56671632	*MT1A* (near-gene 5)	0.047628	0.138
Oxaliplatin	rs562 (C/T)	Chr3:183637845	*ABCC5* (UTR3)	0.049494	0.392
	rs3749445 (A/G)	Chr3:183638506	*ABCC5* (UTR3)	0.044895	0.410
	rs2292998 (C/T)	Chr3:183663833	*ABCC5* (intronic)	0.039882	0.277
	rs1016752 (C/G)	Chr3:183665062	*ABCC5* (intronic)	0.039781	0.232
	rs4148585 (C/T)	Chr3:183670642	*ABCC5* (intronic)	0.039781	0.283
	rs6443924 (A/G)	Chr3:183679532	*ABCC5* (intronic)	0.039781	0.279
	rs4148579 (A/G)	Chr3:183685249	*ABCC5* (intronic)	0.039781	0.279
	rs939336 (A/G => C594C)	Chr3:183685534	*ABCC5* (exonic)	0.039781	0.279
	rs1132776 (C/T => A395A)	Chr3:183696402	*ABCC5* (exonic)	0.044895	0.289
	rs2313212 (C/T)	Chr3:183700928	*ABCC5* (intronic)	0.039781	0.279
	rs4148575 (C/T)	Chr3:183702275	*ABCC5* (UTR3)	0.044895	0.289
	rs1846692 (C/T)	Chr16:56671696	*MT1A* (near - gene 5)	0.049494	0.360
	rs35346959 (A/G)	Chr16:56671867	*MT1A* (near- gene 5)	0.044895	0.137
	rs9922957 (C/G)	Chr16:56672380	*MT1A* (near - gene 5)	0.039781	0.185
	rs9922409 (A/G)	Chr16:56672400	*MT1A* (near - gene 5)	0.04532	0.103
	rs7190725 (G/T)	Chr16:56673290	*MT1A* (intronic)	0.044895	0.170
	rs8052394 (A/G => K51R)	Chr16:56673828	*MT1A* (missense)	0.044895	0.152
	rs1800566 (C/T => P187S)	chr16:69745145	*NQO1* (exonic)	0.039781	0.258
	rs689455 (A/C)	Chr16:69761661	*NQO1* (near- gene 5)	0.039781	0.242

Significant hits from association at FDR corrected threshold of 0.05 are shown in this table. All hits are known dbSNP common variants. All data is reported from Database of Single Nucleotide Polymorphisms (build 137).

**Figure 2:**
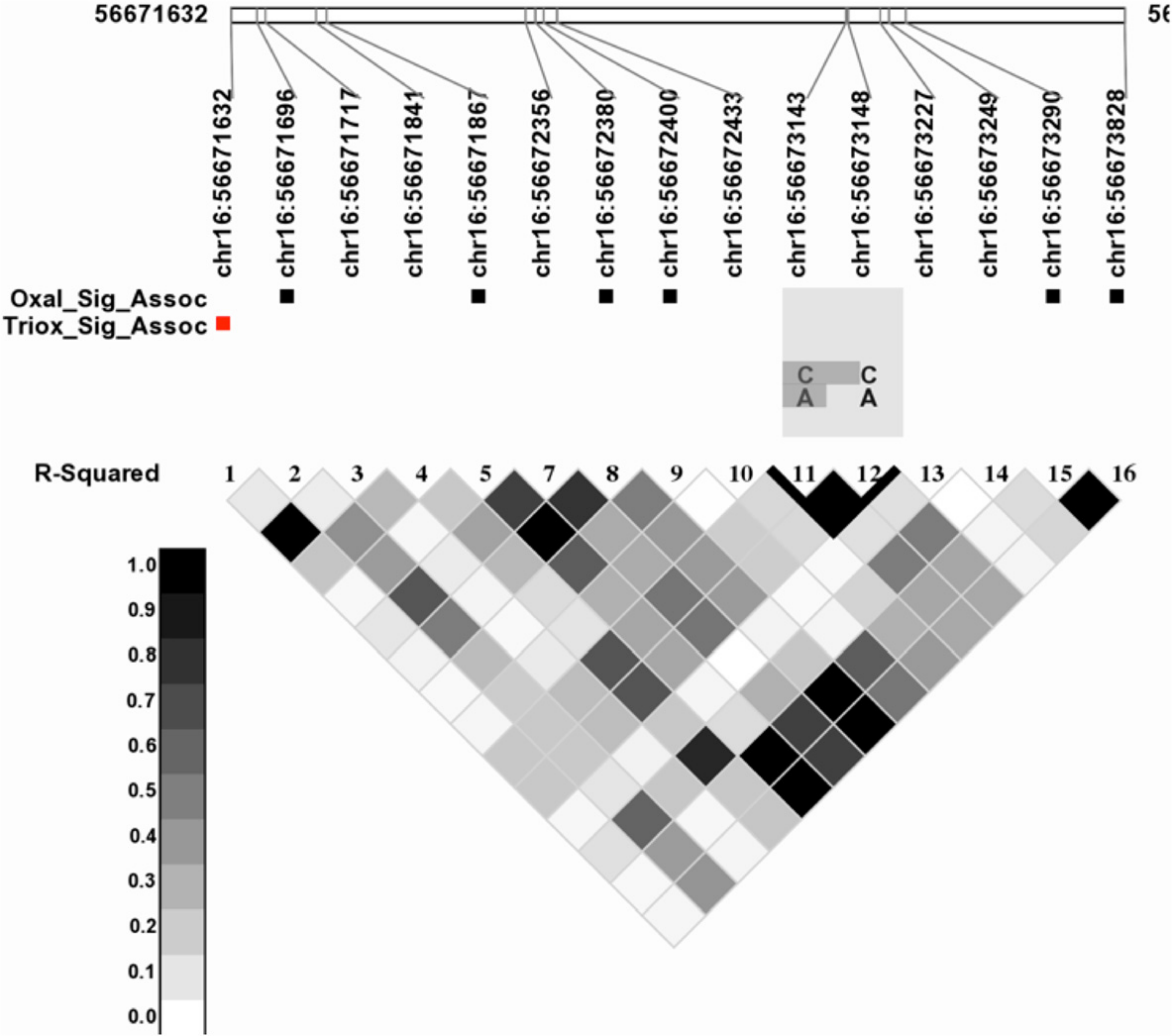
**Linkage disequilibrium plot for MT1A.** Markers significantly associated with Oxaliplatin are highlighted with black boxes and the one associated with Arsenic Trioxide is marked with a red box.

There was a single SNP associated with Arsenic Trioxide response. This SNP (rs1846692) is a known SNP, and is upstream of the gene metallothionein (*MT1A*) on chromosome 16. While this SNP is not in that gene, it is in strong linkage disequilibrium with variants in that gene, as shown in Figure 2.

**Figure 3:**
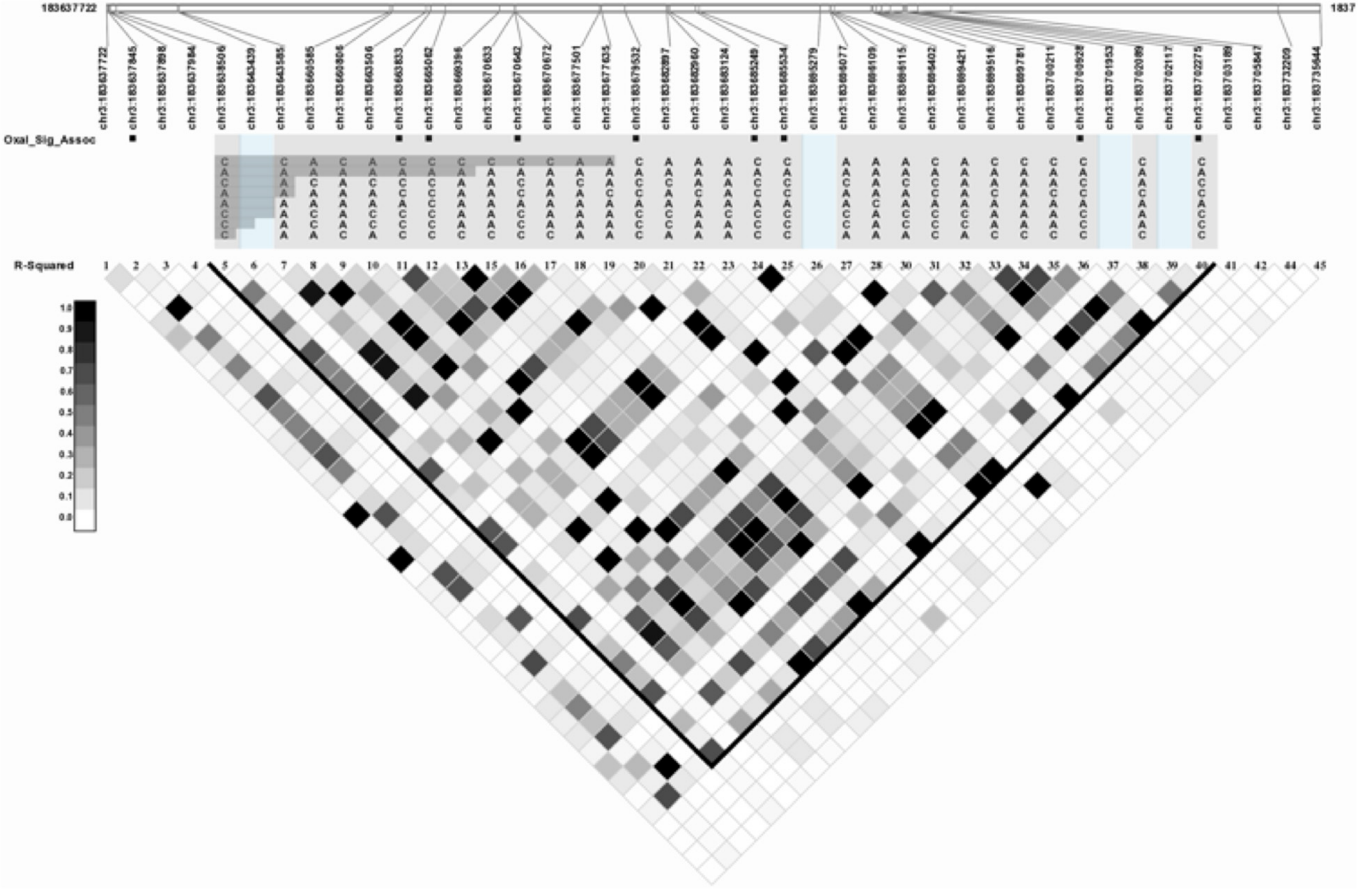
Linkage disequilibrium plot for markers in ABCC5: Markers significantly associated with oxaliplatin are highlighted with black boxes.

**Figure 4:**
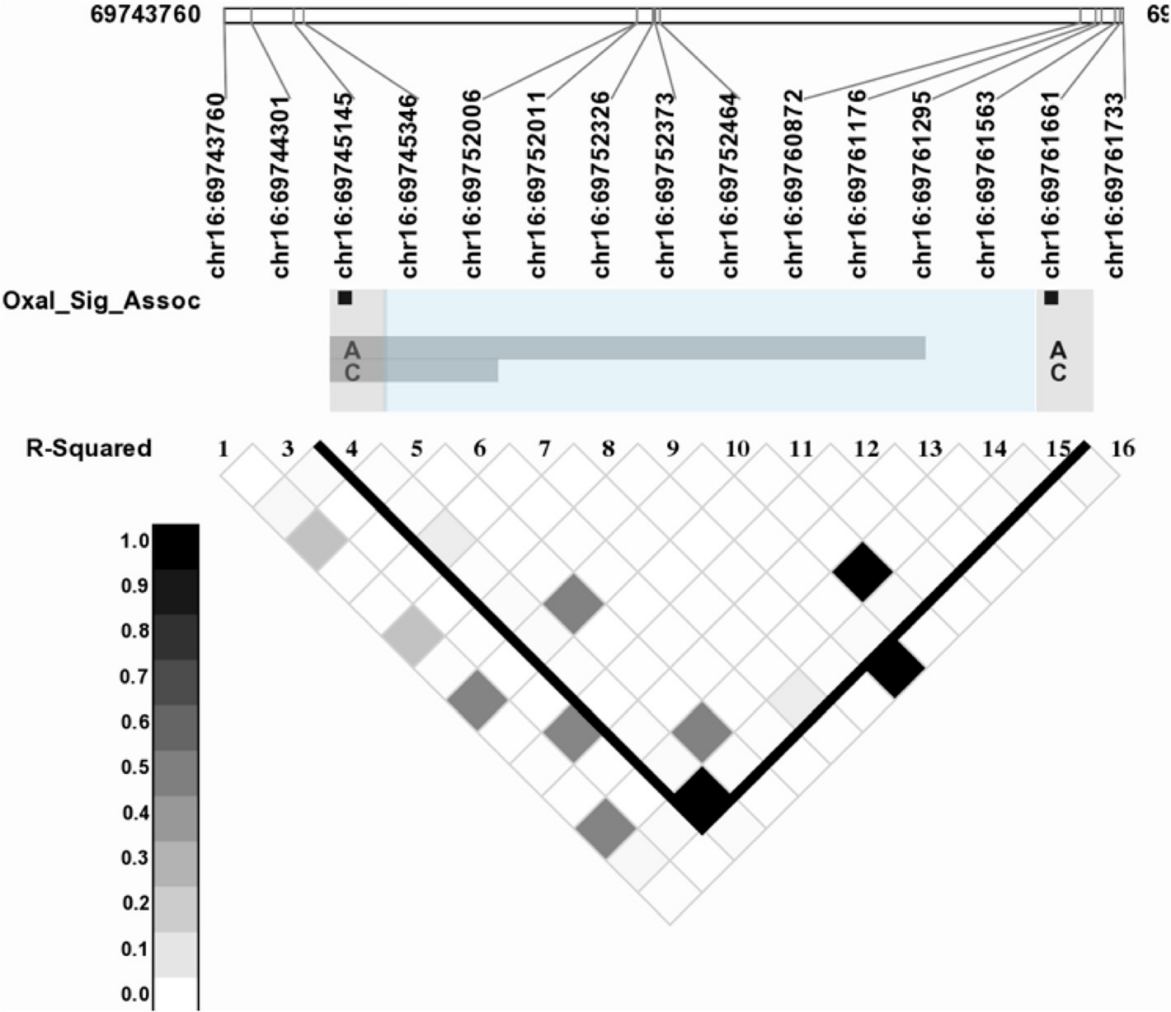
Linkage disequilibrium plot for markers in NQO1: Markers significantly associated with oxaliplatin are highlighted with black boxes.

A total of 19 SNPs were significantly associated with Oxaliplatin response. Of those 19, 6 SNPs are dispersed in introns, exons and near the 5′ region of the gene *MT1A*. Another 11 SNPs are in the untranslated region (UTR3), intronic and exonic regions of the gene ATP-binding cassette, sub-family C, member 5 (*ABCC5/MRP5*) on chromosome 3, and the remaining 2 SNPs are in the gene NAD(P)H dehydrogenase, quinone1 (*NQO1*) gene on chromosome 16. Linkage disequilibrium patterns across these genes are shown in Figures 3 and 4.

These gene associations represent interesting potential drug response genes. *ABCC5* belongs to the class of ATP-binding cassette transporters and is involved in cellular transport of cyclic nucleotides [10]. *ABCC5* has previously been associated with oxaliplatin resistance [11] and other platinum drugs [12]; although the role of *ABCC5* in drug resistance is not clear [13]. In addition to *ABCC5*, variants in *NQO1* including a non-synonymous variant (rs1800566) were associated with oxaliplatin. *NQO1* is a member of the platinum pathway and is involved in metabolism of platinum based drugs. *NQO1* has been linked with resistance to another platinum containing drug cisplatin [14-16] and may share a common role with metabolism of oxaliplatin. This reaffirms the potential of the model to detect clinically meaningful associations, making us more optimistic about the potential relevance of the other findings. While the nature of any candidate gene study is that the genes chosen have biological relevance to the trait of interest, it is important to note that since the candidates were chosen across all 30 drugs in the current study, there is ample opportunity to discover novel associations.

Variants in the metallothionein 1A (*MT1A* gene) were significantly associated with both Oxaliplatin and Arsenic Trioxide. This respresents a novel association with these two drugs to the best of our knowledge. *MT1A* has been previously shown to be associated with cisplatin resistance [17], but it is not known how this relates to Oxaliplatin and Arsenic Trioxide. We did look up expressions quantitative trait loc (eQTL)status for these SNPs using NCBI’s Phenotype-Genotype Integrator (PheGenI) database, and none of this significant SNPs were in known eQTL regions [18]. These associations should be followed up in future studies, in both functional experiments and clinical samples.

## Conclusions

This study selected 14 CEPH LCL pedigrees to perform association analysis of 103 candidate genes to drug response for 30 chemotherapeutics. The 103 candidate regions were sequenced with sufficient depth to determine variants within each LCL. Sequencing data allowed us to have a higher resolution for the markers called than genotyping data from standard genotyping chips would have allowed. The added benefit of detecting more variants than offered by a genotyping chip comes at the cost of time required to set up the pipeline for variant calling (even with open source software), computational time, space and resources required to process each sample and data management. Approximately 2.5TB of data generated from this sequencing study (including raw FASTQs, processed FASTQs, intermediate alignment files, and variant call files) allowed us to call 2281 variable genotypes across the 94 samples.

We conducted FBAT for all the markers discovered in this study for 30 drug response phenotypes. When accounting for all markers and drugs, no hits are significant after an FDR correction. This is not surprising and the loss of power to detect any significant association can be attributed to the small sample size used in this study and a heavy burden of multiple hypotheses testing correction (for 30 drug phenotypes). However, when we do not apply correction for phenotypes but only correct for multiple markers per drug, we observe significant hits for 2 drugs at FDR corrected threshold of 0.05. While our correction strategy is not strictly conservative, we feel that it is appropriate given the exploratory/hypothesis-generation goals of the study.

Only 2 of 30 drugs showed significant hits and no SNPs in known drug metabolizing enzymes were significantly associated with our trait of interest. It is quite possible that none of the markers in these genes had a big enough effect size for other drugs to be easily detected in this study. Also, it is well known that drug response is a complex phenotype and SNP interactions may be able to explain some of the observed variability in drug response; but they were not tested in this study. There is a possibility that mechanisms other than polymorphisms in genes may have an influence on drug phenotype (e.g. miRNA, transcript expression). In conclusion, while the small sample size limits the inference that can be made in the current data, this study is a proof-of-principle demonstration of the use of sequencing data in the cytotoxicity models of human cell lines, and has generated novel gene associations that should be further investigated in future studies. As sequencing data becomes more accessible, such an approach will likely be more commonly applied to associate rare and novel variants alongside common variants with drug response that would have otherwise been missed by GWAS chips.

## Methods

### Cytotoxicity phenotypes

The cytotoxicity data from Peters et al. were used as the phenotypes for association analysis. Briefly, 125 lymphoblastoid cell lines from 14 CEPH families were treated with four doses of each of 30 chemotherapy drugs (A list of drugs used in this study is available in Table 1) to capture the linear portion of the cell kill curve. Cell viability was used as a measure for drug induced cytotoxicity - higher cytotoxicity would result in lower cell viability indicating better drug response; whereas lower cytotoxicity would account to higher cell viability implying poor drug response. The cell viability was quantified using the non-toxic Almar Blue reagent which is converted into a fluorescent compound by the living cells. The fluorescence of drug treated cells was measured relative to cells treated with vehicle control (DMSO) to account for background noise. Outliers were removed and one average measurement across replicates was used for any further analysis. Drug response measured via cell viability was used as the phenotype in our association study. Details of the phenotyping experiments can be found in [5]. Details of the quality control measures used can be found in [19]. Most of the drugs tested showed an estimated heritability of 0.3 or greater in the Peters et al. study [5] indicating that the genetic component explains a significant amount of variability in drug response.

### Selection of cell lines for sequencing

Of the four drug concentrations for each antitumor agent, the one that resulted in an average viability closest to 50% across all cell lines was used for determining sensitivity or resistance to that agent. Cell lines demonstrating extreme responses, defined as viability below the 10^th^ or above the 90th percentiles of the viability distribution at the selected dose for a given drug, were respectively labeled as sensitive and resistant to that drug. Ninety-five CEPH cell lines that displayed sensitivity and/or resistance to at least one of 23 drugs were selected for candidate gene sequencing. The 23 drugs upon which the selection criteria were based included 14 drugs from one of 5 drug classes (fluoropyrimidines, anthracyclines, platinum compounds, taxanes, and camptothecins), as well as an additional 9 drugs for which the cytotoxicity profiles across the entire set of 125 cell lines showed high correlation with those of the 14 targeted drugs. A complete list of the 95 cell lines and pedigree structure is available in Additional file 1: Table S1.

### Candidate gene sequencing

103 candidate genes were selected for resequencing based on their involvement in pathways for drug metabolism, transport, or drug action for 5 classes of chemotherapy drugs tested in the in vitro cytotoxicity assay described above: fluoropyrimidines, anthracyclines, platinum compounds, taxanes, and camptothecins. The candidate genes selected are listed in Additional file 1: Table S2. A multiplex PCR reagent for amplification of the exons, including untranslated regions and approximately 1000 bp upstream of the first exon, from all the candidate genes was designed by RainDance Technology (RDT). This technology allows for independent amplification of multiple PCR reactions in a single tube through the sequestration of primer pairs for each amplicon in separate, picoliter-volume microdroplets [20]. A total of 1932 amplicons were designed to capture the 103 candidate genes. The mean amplicon size was 514 bp (range 206-600 bp), and up to 18 amplicons were tiled to cover large exons. The total amount of genomic DNA sequence expected to be amplified by this PCR multiplex is 800,965 bp. After merging microdroplets containing genomic DNA from each of the 95 cell lines with the multiplex, primer microdroplet mix on an RDT1000 microfluidic station, samples were PCR amplified, amplicon DNA was purified, ligated, randomly sheared, and used to prepare sequencing libraries. Libraries were sequenced on Illumina Genome Analyzer II (GAIIx) sequencers to generate 36 base, single end sequences (i.e., only sequenced one end of the read) using between one and 9 sequencing lanes per sample, resulting in high depths and coverage across the amplicons.

### Pipeline for variant discovery

The sequencing data obtained from different centers was subjected to rigorous data cleaning before variant calling. We received data for all samples in FASTQ format. A FASTQ is a standard file format to store sequence and Phred scaled base quality information [21]. A Phred quality score is the -10*log10(estimated probability that the base was called incorrectly). Every FASTQ (141 for 95 samples) went through the following pipeline that was developed at Expression Analysis Inc, and modified for application to this dataset:

1. Adapter clipping and low quality base trimming: Adapter clipping and read trimming was done using fastq-mcf. Any adapter sequences from the ends of the reads were clipped (adapters are known DNA sequences that allow the DNA molecule to attach to the flowcell where the sequencing chemistry occurs). Also, trailing low quality bases were trimmed from the ends of the reads. Both these steps improve the number of reads that can be mapped back to the genome. FASTQ quality statistics such as nucleotide distribution per cycle and base quality score per cycle were computed to make certain that the sequencing runs had not failed at any cycle and the FASTQs were of high quality.

2. Base Quality Score Recalibration: The Phred base qualities provided by Illumina are known to be inflated for higher quality values [22] and can be corrected by incorporating the error rate from a PhiX control run. A heuristic polynomial model determined from the PhiX alignment data was used to correct for the Illumina provided base quality scores at the clipped FASTQ level. This was done to ensure only high quality variants were called in further analysis.

3. Read Alignment and pileup: Burrows-Wheeler Alignment Tool (BWA) v0.5.9 [23] was used to align reads to human genome (Hg19). Default parameters were used for alignment and generation of the Sequence Alignment Map (SAM) files that contain alignment coordinates and mapping qualities in a standard format [24]. Statistics for the aligned files were generated to gain an idea of the capture quality by quantifying the number of reads mapped, mapping qualities, and percent alignment on different chromosomes. The SAM files were then converted to pileup formats using Samtools v0.1.15 [24] which were consequently used for computing quality statistics. Pileup format gives base-by-base information for all aligned reads in terms of chromosomal position, reference base, the number of reads aligned at that position, base calls, and base qualities [24]. Rigorous quality statistics were computed using the pileup format to assess the PCR and hybrid capture processes. These quality checks include coverage, depth, uniformity of coverage, number of bases that fall inside of amplicons vs. outside of amplicons, number of reads that fall within the amplicons vs. outside of them (unpublished work, Expression Analysis Inc, Durham), and total number of amplicons captured.

4. Variant Calling: Prior to SNP calling, samples with replicate runs were merged into one BAM file (binary equivalent of SAM). Along with the merged bam file, variant calling was conducted on each replicate to allow for consistency check. The bam files were then realigned around regions containing insertions or deletions (indels) to minimize the number of mismatch SNPs and false SNP calls and used for single sample SNP calling. For indel calling, a secondary realignment around known dbSNP indel regions (Available from: http://www.ncbi.nlm.nih.gov/SNP/) was done, followed by variant calling. The realignment and variant calling were done using the open source JAVA software, Genome Analysis Tool Kit (version 1.4-30-gf2ef8d1) (GATK) [25] using the default parameters and all available depth (no downsampling was carried out). Genotype calls provided by GATK were used for association analysis.

5. Variant Filtering: Due to the few number of variant calls made in the candidate regions, the GATK variant filtering could not be used; and custom filters were set up to ensure that highest quality variants passed through. SNPs meeting any of the following criteria were masked to unknown genotypes:

a. Quality By Depth (GATK parameter): <=2.5; This is a GATK parameter which is computed as phred scaled probability of observing a variant at the given site/depth at the site. Low QD values may indicate errors (Broad Institute, 2011)

b. Strand Bias (GATK parameter): > = 60; GATK parameter where high values indicate that mostly one of the strands is showing evidence for the variant.

c. Depth: <=5

d. Known dbSNP indel was called within the individual at this position

e. If any two of the following conditions were met, the genotype calls were masked out:

i. The site under consideration was called with multiple alleles in the given sample of 95 individuals

ii. Additional variant calls were made within a few bases of the site in this individual or the population (Possible indication of alignment error or sequencing error of low complexity region)

iii. The variant call was made within 3 bases to the left of the sense primer or 3 bases to the right of antisense primer

f. Mendelian rules of transmission were used to rescue inaccurately called genotypes in offspring.

6. Variant Quality/Genotype Quality assessment: Genotypes from samples were compared to available Hapmap phase III sample calls (http://hapmap.ncbi.nlm.nih.gov/). Genotypes across different replicates in the candidate gene study were compared. Genotype consistency was also determined by Mendelian error checks. To compute Mendelian error, a superset of variant positions was generated for every nuclear family (trio) in a pedigree. For each variant position, if a variant call was not made in a family member in presence of sufficient depth (20), then a homozygous reference genotype was assigned to that member. Variant Quality assessment was also made in terms of dbSNP membership.

### Association analysis

Family Based Association Testing (FBAT version 2.0.3) software was used to test the null hypothesis of no linkage or no association of marker with unknown trait locus. The testing was conducted for autosomal markers only (sex linked markers were not used in this study). The minimum number of informative families was set to 4 in order to maximize the utilization of available marker set. The offset value for each phenotype was set to the sample mean of that phenotype because the phenotypes were relatively normal and no ascertainment bias from extreme percentiles of cell viability distribution was observed.

### Implementation

The pipeline for variant calling was implemented in bash scripting. Variant filtering was done using custom R and perl scripts. The pipeline was run on a cluster of 30 commodity servers with 4 to 24 CPUs per node, 8 to 60 GB of RAM per node, and Ubuntu Linux as the operating system. To process a single FASTQ with approximately 14M reads for steps 1 – 3 on a single CPU with 40 GB of RAM required 2 hours and 45 min of CPU time. Alignment to the human genome was the most time consuming step (~91 minutes).

### FASTQ quality assessment

Due to processing of DNA samples at four sequencing centers, we had 141 FASTQ files (FASTQs) generated for 95 LCLs with each sample having between 1 to 9 FASTQs. The read lengths and number of reads per FASTQ varied for different samples across centers. The number of reads per FASTQ ranged from 16K to 29M indicating cluster generation problems for certain flowcells. The low count read FASTQs were discarded from any further analysis as an entire flowcell showed cluster generation problems. We were left with 134 FASTQs for the 95 samples.

### SAM quality and Amplicon capture assessment

The FASTQs were processed and aligned to UCSC hg19 assembly using BWA software. For the candidate gene study, 94.08% reads on average (±1.52% sd) aligned per sample with a median mapping quality score of 37. Unsurprisingly, samples with higher number of reads showed higher coverage – more targeted amplicons were sequenced and at higher mean depths. The average depth over all amplicons in a sample was 255.20 (±130.29 sd). The coverage (percent bases across all amplicons covered at least 1X) varied from 85.40 – 99.79%. The high variability in mean depth and coverage can be accounted to low read counts from 3 different flowcells and variability in on-target performance. On-target performance was assayed in terms of percent bases on target vs. outside of targeted regions – on average, 45.66% (±9.43 sd) of total sequenced bases within a sample aligned to the targeted regions.

The uniformity of capture at a given depth was calculated for every amplicon per sample using a measure called Area Under the Reference Line (AURL) (unpublished work, Expression Analysis Inc, Durham), where the reference line is defined by the depth under consideration. For a uniform coverage at that depth, the AURL will be close to 1; non-uniformity resulting from depths lower than reference line reduces the value of AURL towards zero. Overall, the mean AURL within a sample was 94.80 and 92.95 at depths 20 and 30 respectively; signifying that the amplicons within an individual were uniformly covered at sufficient depth to call variants for most positions. The samples that showed low AURLs belonged to flowcells with low read counts. Given the relatively low performance of these lanes; 15 replicate samples were discarded from further analysis without resulting in any data loss for the 95 LCLs.

### Variant calling

The Genome Analysis Toolkit from BROAD Institute was used for variant calling and filters described in Methods section were set to retain high quality variants. Additional file 1: Figure S1 summarizes Mendelian segregation error rates for the trios generated from all pedigrees after SNP variant filtration. One of the family trio (offspring 12142 from pedigree 1334) showed very high Mendelian error rate even after filtering (40.82%) – indicating that the sample might have been mislabeled and was excluded from any further analysis. One other sample (NA12700) that showed high Mendelian error rate before filtering was sequenced over nine lanes of flowcells and variants were called at extremely high depths for this sample, but the error rate substantially reduced (1.76%) after filtering of variants. The remaining trios showed on an average 7.88 ± 4.63% error rates before filtering and 0.87 ± 0.59% after filtering. Hapmap data was available for 18 samples used in this study – this was used to check genotype consistency from sequencing data. Before filtering, an average of 96.41 ± 1.27% of variants in Hapmap were called in sequencing data with matching genotypes and 99.79 ± 0.12% of homozygous reference calls were called non-variant sites in sequencing data. After filtering, an average of 95.32 ± 2.17% of variants in Hapmap were called consistently in sequencing data and 99.88 ± 0.08% of reference calls were called non-variant in sequencing data (we improved specificity as the cost of sensitivity) (Additional file 1: Figure S2). Variant quality was further assessed by looking at dbSNP membership of the calls. Most of the filtered calls were non-dbSNP members (Additional file 1: Figure S2). The various quality assessments ensure that we were left with 2281 high quality SNPs in the targeted regions across all cell lines for association analysis. Genotype calls for >78% SNPs were available in 90 samples or more.

## Competing interests

The authors declare that they have no competing interests.

## Authors’ contributions

GH performed the data analysis and prepared the manuscript. EL, TH, and PK performed the sequencing and conducted the dose response experiments. HM, MW, and AMR planned the study, and contributed to the manuscript preparation. All authors read and approved the final manuscript.

## Supplementary Material

Additional file 1 Supplementary tables and figures. Click here for file
