# Erratum to: Application of next generation sequencing to CEPH cell lines to discover variants associated with FDA approved chemotherapeutics

**DOI:** 10.1186/1756-0500-7-652

**Published:** 2014-09-17

**Authors:** Gunjan D Hariani, Ernest T Lam, Tammy Havener, Pui-Yan Kwok, Howard L McLeod, Michael J Wagner, Alison A Motsinger-Reif

**Affiliations:** Bioinformatics Research Center, North Carolina State University, 307 Ricks Hall, 1 Lampe Dr, 27695 CB7566, Raleigh, NC USA; Department of Statistics, North Carolina State University, Raleigh, NC USA; Cardiovascular Research Institute, UCSF School of Medicine, San Francisco, CA USA; Center for Institute of Pharmacogenomics and Individualized Therapy, UNC Chapel Hill, Chapel Hill, NC USA; Moffitt Cancer Center, Tampa, FL USA

## Correction

After publication of this work [1], it has come to our attention that there is an error in the author list of the initial version of this manuscript; rather than Ernest *J* Lam, the second author of the manuscript should be listed as Ernest *T* Lam.

We publish this correction to update the author list with this information. The correct author list is as follows:

Gunjan D Hariani, Ernest T Lam, Tammy Havener, Pui-Yan Kwok, Howard L McLeod, Michael J Wagner and Alison A Motsinger-Reif

This error was introduced during the final production process and the editors of *BMC Research Notes* apologise for any inconvenience caused.
