# Supplementary material for: Application of next generation sequencing to CEPH cell lines to discover variants associated with FDA approved chemotherapeutics
Source: BMC Res Notes. 2014 Jun 12;7:360. doi: 10.1186/1756-0500-7-360 (PMC4068968; doi:10.1186/1756-0500-7-360)
Supplement: Additional file 1 — Supplementary tables and figures. [file 1756-0500-7-360-S1.docx]

Additional file 1 – Supplementary tables and figures

**Table S.1:** **Pedigree structure for 95 CEPH LCLs.** The 95 CEPH LCLs from 14 families sequenced in the study are listed in this table along with the pedigree structure.

| **Family** | **Sample** | **Relation** |
| --- | --- | --- |
| 35 | 12615 | Father |
| 35 | 12616 | Mother |
| 35 | 12617 | Son |
| 35 | 12618 | Son |
| 35 | 12619 | Daughter |
| 35 | 12620 | Daughter |
| 35 | 12621 | Daughter |
| 35 | 12622 | Daughter |
| 35 | 12623 | Son |
| 45 | 12698 | Father |
| 45 | 12699 | Mother |
| 45 | 12700 | Son |
| 45 | 12702 | Son |
| 45 | 12703 | Son |
| 45 | 12704 | Daughter |
| 45 | 12705 | Son |
| 45 | 12706 | Son |
| 45 | 12849 | Daughter |
| 1334 | 10846 | Father |
| 1334 | 10847 | Mother |
| 1334 | 12138 | Son |
| 1334 | 12139 | Daughter |
| 1334 | 12141 | Son |
| 1334 | 12142 | Son |
| 1340 | 7019 | Mother |
| 1340 | 7027 | Son |
| 1340 | 7029 | Father |
| 1340 | 7040 | Son |
| 1340 | 7062 | Daughter |
| 1340 | 7342 | Son |

Table S.1 continued

| 1340 | 11821 | | Son |
| --- | --- | --- | --- |
| 1341 | 6991 | | Mother |
| 1341 | 7006 | | Daughter |
| 1341 | 7012 | | Daughter |
| 1341 | 7020 | | Son |
| 1341 | 7021 | | Son |
| 1341 | 7044 | | Daughter |
| 1341 | 7048 | Father | |
| 1341 | 7343 | Daughter | |
| 1345 | 7345 | maternal grandmother | |
| 1345 | 7348 | Mother | |
| 1345 | 7357 | maternal grandfather | |
| 1350 | 10855 | Mother | |
| 1350 | 10856 | Father | |
| 1350 | 11822 | Son | |
| 1350 | 11824 | Daughter | |
| 1350 | 11825 | Daughter | |
| 1350 | 11827 | Daughter | |
| 1362 | 10860 | Father | |
| 1362 | 10861 | Mother | |
| 1362 | 11983 | Daughter | |
| 1362 | 11984 | Son | |
| 1362 | 11985 | Daughter | |
| 1362 | 11986 | Daughter | |
| 1362 | 11988 | Daughter | |
| 1362 | 11989 | Daughter | |
| 1408 | 10830 | Father | |
| 1408 | 10831 | Mother | |
| 1408 | 12147 | Daughter | |
| 1408 | 12148 | Son | |
| 1408 | 12149 | Daughter | |
| 1408 | 12150 | Daughter | |
| 1408 | 12151 | Daughter | |
| 1408 | 12157 | Daughter | |
| 1420 | 10838 | Father | |
| 1420 | 10839 | Mother | |

Table S.1 continued

| 1420 | 11999 | Daughter |
| --- | --- | --- |
| 1420 | 12001 | Daughter |
| 1420 | 12002 | Daughter |
| 1420 | 12007 | Son |
| 1447 | 12752 | Father |
| 1447 | 12753 | Mother |
| 1447 | 12754 | Daughter |
| 1447 | 12756 | Son |
| 1447 | 12765 | Son |
| 1451 | 12766 | Father |
| 1451 | 12768 | Son |
| 1451 | 12770 | Daughter |
| 1451 | 12771 | Son |
| 1451 | 12772 | Daughter |
| 1451 | 12773 | Daughter |
| 1451 | 12774 | Son |
| 1451 | 12848 | Daughter |
| 1454 | 12802 | Mother |
| 1454 | 12803 | Daughter |
| 1454 | 12805 | Son |
| 1454 | 12806 | Son |
| 1454 | 12807 | Daughter |
| 1454 | 12810 | Son |
| 1459 | 12864 | Father |
| 1459 | 12866 | Son |
| 1459 | 12868 | Daughter |
| 1459 | 12869 | Daughter |
| 1459 | 12870 | Son |
| 1459 | 12871 | Son |

**Table S.2:** **List of 103 candidate genes sequenced in the study.** The genes were selected based on their involvement in pathways for drug metabolism, transport, or drug action for 5 classes of chemotherapy drugs: fluoropyrimidines, anthracyclines, platinum compounds, taxanes, and camptothecins.

| **Gene Symbol** | | | |
| --- | --- | --- | --- |
| MTHFR | POLH | MT1A | UGT2B15 |
| AKR1A1 | REV3L | CES2 | UGT2B7 |
| DPYD | PMS2 | NQO1 | ABCG2 |
| GSTM1 | POLM | TP53 | NFKB1 |
| UCK2 | UPP1 | TOP2A | DHFR |
| FASLG | ABCB1 | MAPT | PSMC1 |
| PARP1 | CYP3A5 | ABCC3 | DUT |
| RRM2 | CYP3A4 | NME1 | ERCC4 |
| XDH | POLB | NME2 | ABCC1 |
| CYP1B1 | GGH | MPO | ABCC6 |
| MSH2 | DPYS | FDXR |  |
| ERCC3 | ACO1 | NT5C |  |
| CFLAR | XPA | TK1 |  |
| UGT1A8 | SLC31A1 | TYMS |  |
| UGT1A10 | FPGS | RALBP1 |  |
| UGT1A9 | AKR1C3 | XRCC1 |  |
| UGT1A7 | ERCC6 | ERCC2 |  |
| UGT1A6 | CYP2C8 | ERCC1 |  |
| UGT1A5 | ABCC2 | PNKP |  |
| UGT1A4 | RRM1 | TOP1 |  |
| UGT1A3 | SMPD1 | SOD1 |  |
| UGT1A1 | GSTP1 | CBR1 |  |
| DTYMK | SLCO1B1 | CDC45L |  |
| MLH1 | TUBA1B | GSTT1 |  |
| MAP4 | UNG | UPB1 |  |
| GPX1 | TUBA3C | TYMP |  |
| NR1I2 | HMGB1 | ATP7A |  |
| UMPS | ATP7B | SMARCA1 |  |
| BCHE | ABCC4 | CSAG2 |  |
| TUBB | MT2A | SLCO6A1 |  |
| ABCC5 | TDP1 | CES1 |  |

**
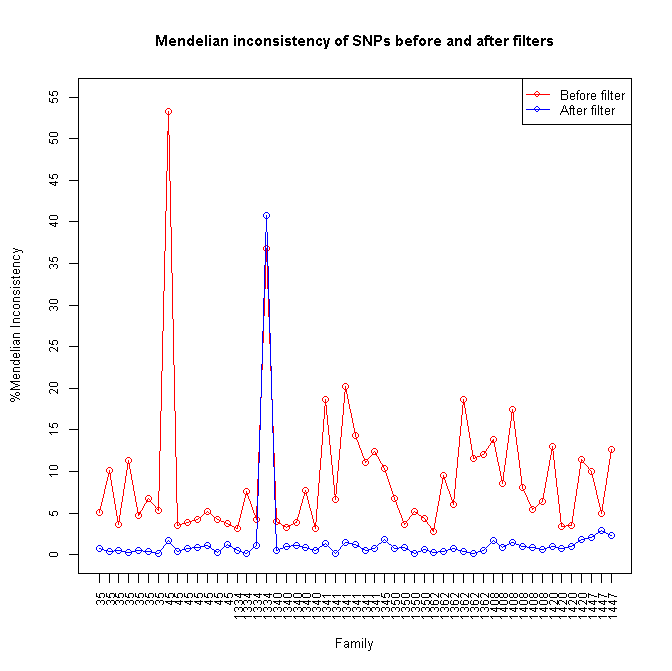
Figure S.1: Mendelian error rates across trios.** Quality of SNPs before and after filtering in terms of Mendelian consistency is shown. Percentage of SNPs showing Mendelian inconsistency in every trio – the inconsistency within all trios was below 3% after filtering.


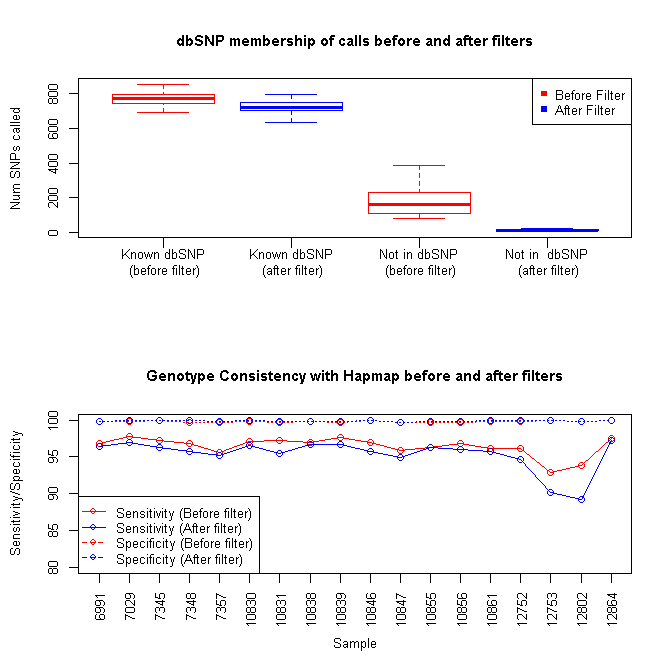


**Figure S.2: Quality statistics for filtered SNPs.** Quality of SNPs before and after filtering in terms of dbSNP membership and Hapmap genotype consistency is shown. The top panel shows the dbSNP membership of SNPs that were retained – a significant decrease in non-dbSNP variants is seen. The bottom panel shows genotype consistency with Hapmap data available for 18 samples. Sensitivity is defined as the percent of variant genotype calls made in Hapmap data, which were also made in sequencing data. Specificity is defined as the percent of homozygous reference calls in Hapmap data that were called non-variant in sequencing data.
